# Discovery of an Inhibitor of Z-Alpha1 Antitrypsin Polymerization

**DOI:** 10.1371/journal.pone.0126256

**Published:** 2015-05-11

**Authors:** Valerie Berthelier, Jason Brett Harris, Kasey Noel Estenson, Jerome Baudry

**Affiliations:** 1 Department of Medicine, University of Tennessee Health Science Center—Graduate School of Medicine, Knoxville, Tennessee, United States of America; 2 UT-ORNL Graduate School of Genome Science and Technology, University of Tennessee, Knoxville, Tennessee, United States of America; 3 Department of Biochemistry and Cellular and Molecular Biology, University of Tennessee, Knoxville, Tennessee, United States of America; 4 UT-ORNL Center for Molecular Biophysics, Oak Ridge National Laboratory, Oak Ridge, Tennessee, United States of America; Computational Biophysics, GERMANY

## Abstract

Polymerization of the Z variant alpha-1-antitrypsin (Z-α1AT) results in the most common and severe form of α1AT deficiency (α1ATD), a debilitating genetic disorder whose clinical manifestations range from asymptomatic to fatal liver and/or lung disease. As the altered conformation of Z-α1AT and its attendant aggregation are responsible for pathogenesis, the polymerization process *per se* has become a major target for the development of therapeutics. Based on the ability of Z-α1AT to aggregate by recruiting the reactive center loop (RCL) of another Z-α1AT into its s4A cavity, we developed a high-throughput screening assay that uses a modified 6-mer peptide mimicking the RCL to screen for inhibitors of Z-α1AT polymer growth. A subset of compounds from the Library of Pharmacologically Active Compounds (LOPAC) with molecular weights ranging from 300 to 700 Da, was used to evaluate the assay’s capabilities. The inhibitor S-(4-nitrobenzyl)-6-thioguanosine was identified as a lead compound and its ability to prevent Z-α1AT polymerization confirmed by secondary assays. To further investigate the binding location of S-(4-nitrobenzyl)-6-thioguanosine, an *in silico* strategy was pursued and the intermediate α1AT M* state modeled to allow molecular docking simulations and explore various potential binding sites. Docking results predict that S-(4-nitrobenzyl)-6-thioguanosine can bind at the s4A cavity and at the edge of β-sheet A. The former binding site would directly block RCL insertion whereas the latter site would prevent β-sheet A from expanding between s3A/s5A, and thus indirectly impede RCL insertion. Altogether, our investigations have revealed a novel compound that inhibits the formation of Z-α1AT polymers, as well as *in vitro* and *in silico* strategies for identifying and characterizing additional blocking molecules of Z-α1AT polymerization.

## Introduction

Human α1-antitrypsin (α1AT) is the most abundant member of the serine protease inhibitor (SERPIN) family. It is a soluble 52-kDa glycoprotein synthesized primarily by hepatocytes and delivered to the lungs to accomplish its critical function: inactivation of the proteinase neutrophil elastase (NE), a mediator of alveolar destruction [1]. Defective folding, trafficking and secretion into the plasma of α1AT are responsible for α1AT deficiency (α1ATD) [2,3].

The structural flexibility of α1AT is important for it to perform its anti-protease function and ensure lung integrity. With a core domain composed of 3 β-sheets A, B and C, and 9 α-helices, α1AT features an exposed and flexible reactive center loop (RCL) that serves as bait for NE. Upon binding to the proteinase, a dramatic conformational change occurs as RCL is cleaved and translocates into β-sheet A to form the new central and fourth strand, s4A. The translocation event carries along NE from one side to the other of α1AT, causing its inactivation by forming an irreversible, higher molecular weight suicide complex [4,5]. A reduction or lack of this inhibition through loop-sheet insertion and proteolytic cleavage is thought to be the underlying mechanism responsible for α1ATD [6,7].

Over 100 genetic variants of α1AT have been identified with the Z-type being responsible for the most common and severe form of the disease in homozygous patients [8]. The point mutation E342K in Z-α1AT renders the anti-protease prone to aggregation and unable to be secreted into the blood stream resulting in a 90% decrease in NE inhibition within the lungs. Accumulation of polymers of Z-α1AT in the endoplasmic reticulum (ER) of hepatocytes leads to proteotoxic stress and associated liver diseases [9–11]. In addition to sequestration of polymers in the ER of hepatocytes, the E342K mutation has two additional disease-causing effects. It causes Z-α1AT to be 5-fold less effective in accomplishing its inhibitory function [12,13] and it promotes the spontaneous formation of Z-α1AT polymers within the lungs, thereby further reducing the already depleted levels of α1AT that are available for alveola protection.[14] Moreover, the conversion of Z-α1AT from a monomer to a polymer renders it a chemoattractant for human neutrophils [15,16]. To summarize, emphysema associated with Z-α1ATD results from a combination of (1) loss of function of the anti-protease, which leads to the absence of circulating α1AT, decrease of its inhibitory activity, and intra-alveolar polymerization, and (2) gain of toxic function from the neutrophil chemotactic properties of intra-alveolar polymers.

Preventing formation and accumulation of Z-α1AT polymers could be crucial to treat α1ATD [17]. For this reason the mechanisms by which Z-α1AT form polymers have been under intense investigation. As the substitution of the glutamic acid residue at position 342 by a lysine provokes a perturbation in the native structure by opening the β-sheet A, biochemical evidence reveals the formation of an unstable and polymerogenic intermediate M* with its own RCL partially inserted [18]. The opening of the s4A cavity allows the creation of a sequential β-strand linkage between the RCL of one serpin and β-sheet A of another, leading to the formation of a dimer and then polymers [6,19–21]. Two additional models for Z-α1AT polymerization have been recently proposed, based on X-ray crystallography experiments, suggesting that assembly pathways of Z-α1AT could be diverse and therefore arising from structurally and/or dynamically distinct polymerogenic intermediates. These models are the s4A/s5A model obtained from a Gnd HCl-induced dimer of a related serpin, antithrombin [22], and the C_term_ swap model based on a heat-induced trimer of a disulfide mutant of α1AT [23]. However, in addition to these alternative mechanisms of polymerization obtained after Gnd-HCl or heat induction, it should be noted that the s4A/s5A polymers are not recognized by a conformation-specific monoclonal antibody of pathological α1AT polymer (Ab 2C1) [23–25].

Various strategies have been pursued in order to prevent or even attenuate Z-α1AT polymerization such as increasing the mutant protein secretion with the use of osmolytes [26–28], or blocking Z-α1AT polymerization by either filling the s4A cavity with peptides [18] or crowding the hydrophobic side pocket of Z-α1AT with small compounds screened virtually [29]. While extensive progress has been made, none of these strategies has been entirely successful so far. To achieve this goal, we developed a set of novel and integrated *in vitro* and *in silico* screenings methods; the *in vitro*, being a high-throughput screening assay using a modified small peptide previously reported as a s4A cavity filler [18], and the *in silico* being a virtual docking model able to predict and help rationalize the binding of compounds to α1AT, including in the s4A cavity. Here, we present how using these two combined methods we were able to identify, rationalize and confirm S-(4-nitrobenzyl)-6-thioguanosine as an inhibitor of Z-α1AT polymerization.

## Material and Methods

### General Materials and Methods

The peptide acetyl-FLEAIGGG-Q-GKKG containing the 6-mer sequence of the RCL was synthesized by custom solid-phase from the Keck Biotechnology Center at Yale University (http://info.med.yale.edu/wmkeck/). A biotinylated version of the peptide (bPEG-peptide) was obtained by appending a biotinyl-polyethylene glycol spacer on the γ-amide group of the glutaminyl residue. The presence of the Lys residues confer a positive net charge to the peptide at neutral pH, enhancing its general solubility. The wild type and Z-α1AT proteins, prepared according to published protocol, [30] were graciously provided at a concentration of 1 mg/ml by Professor Lomas, Cambridge Institute for Medical Research, University of Cambridge, UK, and stored at 4°C.

The rabbit anti-human α1AT antibody (serum fractions IgG) was purchased from Abcam, Cambridge, MA.

The test group RK-001 of the LOPAC library (Library of Pharmacologically Active Compounds, Sigma-RBI, Natick, MA) containing 80 lyophilized chemical compounds was prepared in a 96-well plate format. All compounds were resuspended in 2 ml DMSO at a concentration of approximately 4 mM, based on an estimated MW average of 500 g/moles, and stored at 4°C.

### Preparation of the bPEG-peptide

The synthesized bPEG-peptide was first solubilized in 50% formic acid at a concentration of ~1mg/ml, injected onto a Zorbax C3 Column and purified by RP-HPLC at a rate of 4ml/min. The resulting purified peptide was then lyophilized, resuspended into H_2_O and stored at -20°C. After amino acid analysis of the peptide (Commonwealth Biotechnologies Inc., Richmond, VA), various amounts were injected onto RP-HPLC in order to establish a standard curve, allowing us to determine the exact concentration of each new batch of purified peptide that we prepared.

### Preparation of Working Compound Plates

LOPAC compounds were transferred from their original 96-well plates to new 96-well working plates with low evaporation lid (BD Falcon plates non treated, Becton Dickinson Labware, San Jose, CA) in respect to their initial location, and adjusted to a concentration of 1 mM in PBS 1X containing 50% DMSO. First and last columns were filled only with PBS 1X/DMSO (50/50). Working plates were sealed with an adhesive overlay, covered and stored at 4°C until further utilization.

### Set up of the Microplate Screening Assay

The assay is based on the principle of a competitive ELISA [31]. Wells were coated by passive adsorption with a 1/1000 solution in PBS 1X of capture α1AT Ab. The screening microplate was sealed with an adhesive overlay and incubated for 2 h at 37°C. The wells were then washed three times with extension buffer (PBS 1X and 0.01% Tween 20), blocked for 1 h at 37°C with 0.3% gelatin and washed again. Screening results described in this paper were carried out with screening microplates freshly made. However, screening microplates can be filled with PBS 1X, hermetically sealed and stored at 4°C for one week prior to use.

### Z-α1AT Polymerization Inhibition Assay

In parallel with the preparation of the microplate screening assay, polymerization reactions were carried out in 96-well plates with or without the LOPAC small compounds. A 100 molar excess of bPEG-peptide was used with 4 μg/well of Z-α1AT.

Each polymerization reaction plate was organized as follows: the first column contained only bPEG-peptide (background control); the second to eleven columns contained Z-α1AT, compounds and bPEG-peptide; and the last column contained Z-α1AT, bPEG-peptide and no compound (reaction control). Assay wells were set up by adding to each well 20 μl of protein and 20 μl of compound from a working plate. After 3 min, 160 μl of bPEG-peptide at 48 μM was added. The plate was then sealed, shaken on a microplate shaker gently for 5 s to ensure homogeneity of the different reactants, and placed at 37°C for 16 h. All wells contained 5% DMSO.

At the end of the 16 h incubation time, 100 μl from each well were transferred into the corresponding well of the microplate screening assay. One hour later, the screening plate was washed three times and then incubated in the dark for 1 h at room temperature with 100 μl/well of 1 ng/μl of europium streptavidin (Perkin Elmer, Boston, MA) in 0.5% BSA-extension buffer. Three final washes in extension buffer were carried out and the europium was released from streptavidin by the addition of 100 μl of enhancement solution (Perkin Elmer). After 5 min, europium fluorescence was measured by time-resolved fluorometry in a Victor 2 counter (Perkin Elmer) and then converted to fmoles of bPEG-peptides recruited into Z-α1AT. Assays were conducted in triplicate by processing three identical plates in parallel.

### Determining IC_50_ Values of Inhibitors

As described above, 4 μg/well of Z-α1AT were incubated for 3 min with various concentrations of a compound identified as an inhibitor, the highest concentration starting at 400 μM. The concentrations of the compounds were revised according to the true MW of the molecule. Following the 3 min incubation, 160 μl of 48 μM bPEG-peptide were added and the rest of the protocol was applied as described above.

### Z-α1AT Polymerization Sedimentation Assay

A solution of 0.1 mg/ml of Z-α1AT in PBS 1X was incubated at 37°C with or without 100 μM of S-(4-nitrobenzyl)-6-thioguanosine. Progress of the polymerization reaction was followed by quantitative RP-HPLC on centrifugation supernatants (20 min, 20,000 × *g*) of reaction aliquots. Quantitative determination of the Z-α1AT monomer disappearance was calculated according to a pre-established standard curve.

### Preparing Protein Structures for Homology Modeling and Docking Simulations

The crystal structures of the mutant Z-α1AT (PDB code: 3T1P) and the wild type M-α1AT (PDB codes: 3CWM and 1QLP) were obtained from the RCSB Protein Database [23,32,33]. Using the program MOE (Molecular Operating Environment) [34], co-crystallized water molecules were deleted from both structures. For the polymerized mutant (3T1P), only the first monomer was retained and the s4A binding cavity was created between the s3A/s5A by deleting residues 345–356, which correspond to the inserted residues of the RCL. The protonation state of atoms was assigned using Protonate 3D [35] utility in MOE at pH 7, 300 K and 0.1 M salt concentration. Solvent effects were implicitly included by using a distance-dependent dielectric function. Partial charges were assigned to receptor atoms using MMFF94s [36] force field parameters as implemented in MOE.

### Homology Modeling Procedure

Modeling of the M* intermediate state was performed using the Homology Model facility in MOE that allows the building of a model utilizing multiple template structures simultaneously. To generate the M* intermediate model, two distinct template structures of α1AT (PDB codes 1QLP and 3T1P) were used. These two template proteins are close matches of each other with 98.6% sequence identity, differing most notably by a mutation replacing Glu 342 (structure 1QLP) by Lys 342 (structure 3T1P), which distinguishes the wild-type protein from the polymerizing Z mutant variant. Due to the dynamic nature of α1AT, the two PDB models represent distinct stable-state structures of the protein. The wild type (1QLP) possesses a closed β-sheet A with the RCL extended along the outer edge of the protein, followed by the C_term_ loop stably inserted in β-sheet B; whereas, the mutant structure (3T1P) has an expanded β-sheet A with the RCL inserted and the C_term_ loop of one monomer extended in order to domain swap with β-sheet B of a neighboring monomer. Therefore, the 1QLP crystal structure was used as a template for modeling i) the position of the RCL when not inserted into β-sheet A and ii) the position of the C_term_ loop within β-sheet B when it is not participating in a domain swap. The 3T1P mutant structure was used to model the expanded position of β-sheet A while omitting s4A in order to leave a cavity between s3A/s5A where the RCL would otherwise be found. Fragments from each template structure were joined at transition points selected by superimposing the template structures and choosing those residues between fragments with near overlapping atom positions.

Since proteins inherently have many degrees of freedom, finding a single representative model is a challenge often requiring sampling many possible conformational states. MOE allows for sampling an ensemble of possible structural models, not unlike molecular dynamics, using conformational sampling techniques along with hierarchical rounds of geometric and energetic model refinement until a stable low energy model is identified to represent the ensemble of structures. In the case of sampling possible M* intermediate state models, a total of 25 initial models were first generated each with unique carbon backbone positions. For each of those 25 models, 5 additional models (*i*.*e*. a total of 125 models) were created with alternate side chain positions in order to provide sampling of possible side chain degrees of freedom. These initial models were then energy minimized to a gradient of 0.1 kcal/mol·Å. A final M* model (Model 126) was created using the ensemble of structures and the Generalized Born / Volume Integral (GB/VI) energy scoring method [37] to select the best initially packed structure and then the structures were further energy minimized to a gradient of 0.01 kcal/mol·Å.

### Docking Simulation Procedure

Three-dimensional structures of the 80 *in vitro* tested chemicals, including S-(4-nitrobenzyl)-6-thioguanosine, were obtained in SDF format from the electronic LOPAC library, test group RK-001. The MMFF94s force field was used to assign parameters and partial atomic charges to each ligand. Energy minimization of the structures was performed for each ligand to a gradient of 0.1 kcal/mol·Å. The energy-minimized ligands were docked into three structural variations of α1AT: M* intermediate (Homology Model 126), mutant Z-α1AT (PDB: 3T1P) and wild type M-α1AT (PDB: 3CWM) using the docking facility built into MOE. Using the MOE Site Finder facility, several likely binding sites were identified for each of the respective receptor models (wild type, mutant, and M* intermediate). This tool uses polar and nonpolar spheres (1.4 Å and 1.8 Å in radius, respectively), to sample the protein surface for nonpolar or polar contact points and saves locations of the protein as ‘binding sites’ if they contain three or more adjacent spheres making favorable contact with the protein surface. The sites identified for each receptor type are listed in Table 1. Docking calculations of the 80 compounds were carried out separately for each of the three receptor structures and at each potential binding site. During docking, initial placement of ligand atoms in each potential binding site used the Triangle Placement method. The binding free energy of each pose using the London dG [34] scoring method. The top five scoring poses were further energy minimized using the MMFF94s force field, allowing ligand atoms and protein side chains within 6 Å of each docked ligand to be treated as flexible. A tethering weight of 10 kcal/mol/Å^2^ was applied to partially restrain flexible atoms around their original location. A final docking score / binding free energy estimate for each energy-minimized pose was calculated using the Affinity dG [34] scoring method.

**Table 1:** Docking results from M-α1AT, Z-α1AT, and M* model with S-(4-nitrobenzyl)-6-thioguanosine and the 79 other small molecules.

Structure	SITE	Lowest Energy^β^ (kcal/mol)	B9 Energy^δ^ (kcal/mol)	B9 Rank^γ^
M-α1AT (3CWM)	SITE1	n/a^Θ^	n/a	n/a
	SITE2	-7.0	-5.6	19^th^
	SITE3	-6.2	-5.3	8^th^
	SITE4	-6.4	-3.4	25^th^
	SITE5	n/a	n/a	n/a
	SITE6	-7.6	-7.6	1^st^
Z-α1AT (3T1P)	SITE1	n/a	n/a	n/a
	SITE2	-6.5	-3.9	34^th^
	SITE3	-9.0	-5.8	13^th^
	SITE4	-4.6	-3.6	19^th^
	SITE5	n/a	n/a	n/a
	SITE6	n/a	n/a	n/a
M* Model	SITE1	-10.7	-7.9	7^th^
	SITE2	-10.7	-8.8	5^th^
	SITE3	-5.1	-4.6	9^th^
	SITE4	n/a	n/a	n/a
	SITE5	-8.4	-8.4	1^st^
	SITE6	-6.2	-4.22	12^th^

^α^ Site number where S-(4-nitrobenzyl)-6-thioguanosine (B9) was docked.

^β^ Lowest observed binding energy (kcal/mol) for any of the 80 docked compounds.

^δ^ Predicted binding energy (kcal/mol) for B9.

^γ^ Rank of B9 relative to the binding energies for all 80 docked compounds.

^Θ^ Site numbers that are not found in a given model are noted by a not applicable symbol (n/a).

## Results and Discussion

### Characteristics of the Z-α1AT Polymerization Inhibitor Screening Assay

Previous studies have shown that a 6-mer peptide whose amino acid sequence contains the RCL sequence FLEAIG can specifically bind the Z-mutant at its opened s4A pocket, but not the wild type [18]. The Z-α1AT high-throughput microplate screening assay is based on this concept. Thus, we designed a similar peptide and added a biotin-polyethylene glycol (bPEG) tag at the C_term_ of the reactive loop sequence as well as some hydrophilic amino acids to increase peptide solubility. The insertion of a PEG-based spacer prevents possible steric hindrance between the peptide and the biotin molecule, resulting in better avidin binding and therefore, a more accurate measurement of the biological activity.

To assess the ability of small molecules to inhibit the recruitment of the bPEG-peptide into Z-α1AT, Z-α1AT is subjected for 3 min to library compound before addition of bPEG-peptide and the resulting complex formed is transferred into microtiter wells containing the attached antibody. The amount of bPEG-peptides incorporated into the mutant protein is then determined by a europium-streptavidin treatment and time-resolved fluorescence measurements. The inhibition effect of a compound is calculated as a percentage with respect to a reaction control—i.e. Z-α1AT that has only been exposed to the biotinylated peptide and not to a compound. Any compound showing an inhibitory effect of at least 50% is considered as a hit.

**Fig 1:**
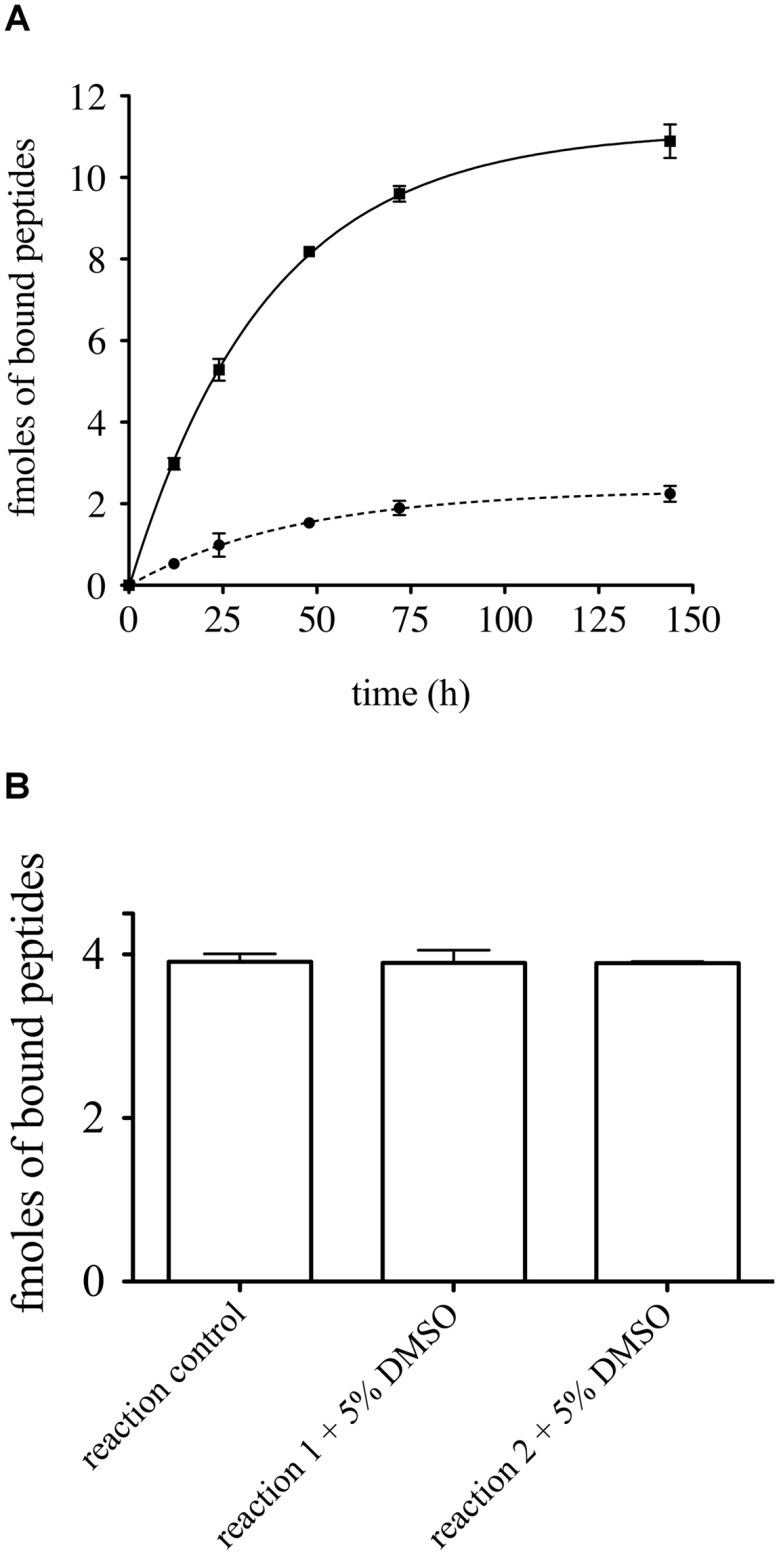
Kinetic diagram of bPEG-peptide binding to α1AT. (A) Four micrograms per well of attached (■) Z-α1AT or (●) M-α1AT were incubated for various times in presence of 38.4 μM bPEG-peptide. (B) Z-α1AT was incubated in presence of 5% DMSO and bPEG-peptide for 16 h. Errors bars reflect the standard deviation of three replicates.

Regarding its ability to bind Z-α1AT, we found the bPEG-peptide association kinetics to be in favor of the mutant proteinase with an initial association rate of 0.22 ± 0.08 fmoles·h^-1^ *vs*. 0.042 ± 0.1 fmoles·h^-1^ for the wild type (Fig 1A). We also found that an incubation period of 16 hrs for the peptide with Z-α1AT is an adequate screening end-point for the screening assay as this time period is associated with a high signal-to-noise ratio. In addition, the presence of 5% DMSO in the wells does not affect the bPEG-peptide binding kinetics (Fig 1B). Since compound libraries are generally stored in DMSO, this feature makes the assay well suited for a high-throughput screening assay.

**Fig 2:**
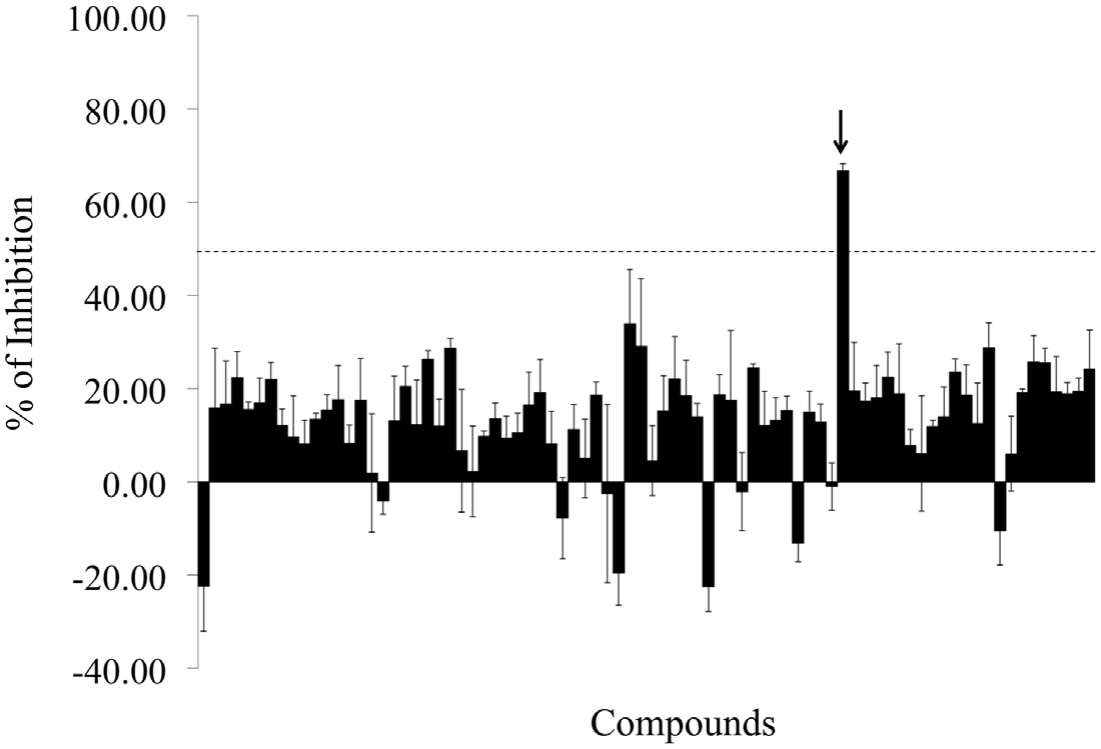
Pattern of inhibition resulting from the screening of 80 unknown LOPAC compounds. A 96-well plate was coated with 4 μg/well of Z-α1AT and incubated for 16 h with 100 μM of various compounds and 38.4 μM of bPEG-peptide. The black arrow indicates the compound that corresponds to S-(4-nitrobenzyl)-6-thioguanosine and gives an inhibition effect of 67 ± 2% and. The error bars are the standard deviation of three individual experiments.

Finally, this screening assay exhibits very good reproducibility as reflected by the error bars shown in Fig 2. It requires only small amount of protein and low concentrations of bPEG-peptide, which make it both economical and physiological.

### S-(4-nitrobenzyl)-6-thioguanosine Identified as Inhibitor of Z-α1AT Polymerization

**Fig 3:**
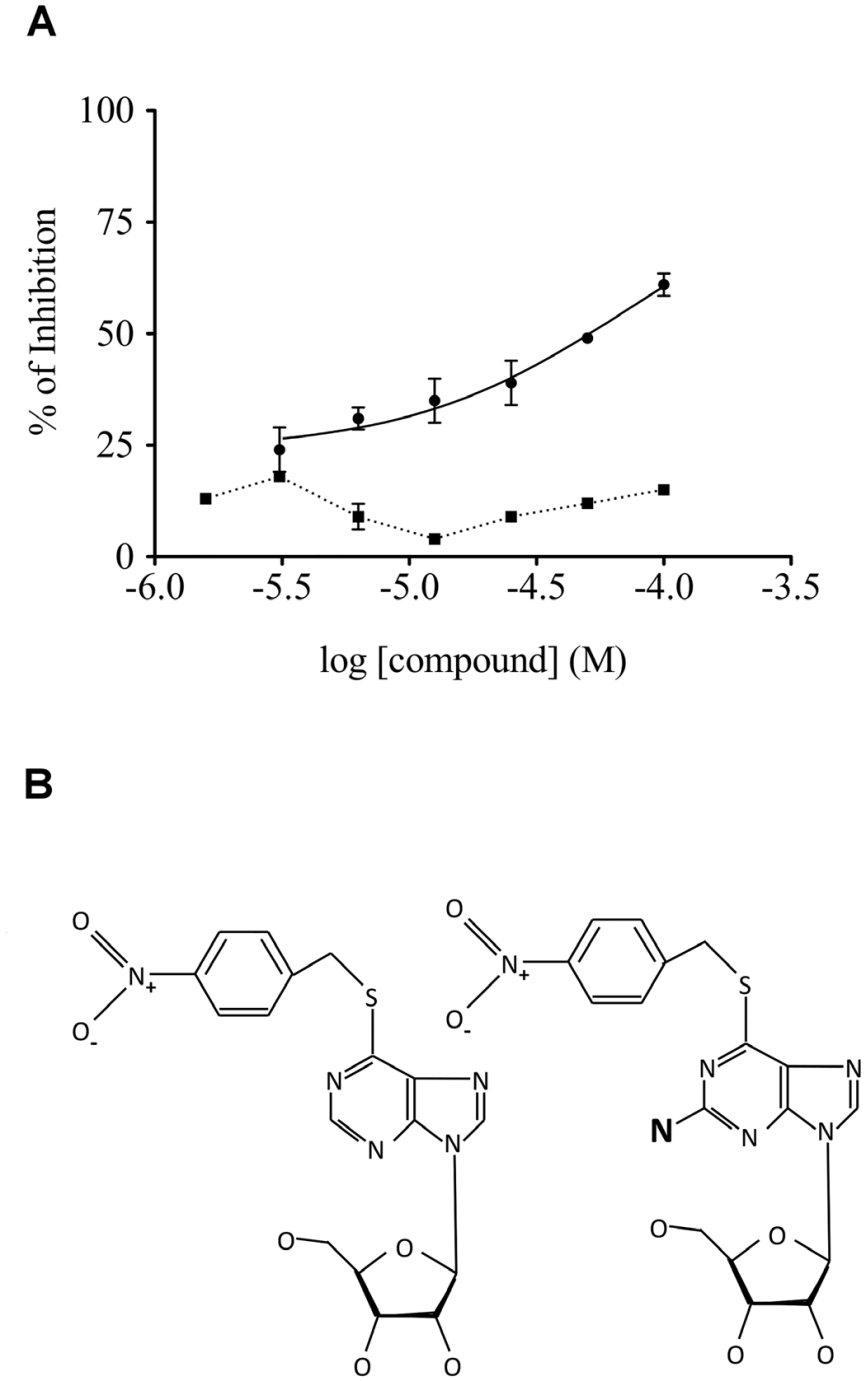
S-(4-nitrobenzyl)-6-thioguanosine inhibits bPEG-peptide binding to Z-α1AT. (A) Dose-response curves were assayed for various concentrations of (●) S-(4-nitrobenzyl)-6-thioguanosine and (■) its homologue S-(4-nitrobenzyl)-6-thioinosine. (B) Chemical structures of (left) S-(4-nitrobenzyl)-6-thioguanosine and (right) S-(4-nitrobenzyl)-6-thioinosine. The errors bars are the standard deviation of an experiment conducted in triplicate.

The test group RK-001 (80 compounds) of the small commercially available LOPAC library containing drug-like molecules was used to test the performance of the screening assay. Fig 2 shows a typical screening result. As indicated in the figure, only one compound of the tested compound plate appears as a hit, exhibiting a 67 ± 2% inhibition activity at 100 μM. This compound is S-(4-nitrobenzyl)-6-thioguanosine. To confirm its ability to inactivate Z-α1AT polymerization, dose-responses curves were carried out and an IC_50_ of 73 ± 0.12 μM calculated (Fig 3A). The IC_50_ value obtained is in the micromolar range and matches well with the screening results.

To define a better pharmacophore, and therefore to identify any additional structural element required for inhibiting Z-α1AT polymerization, we then compared our compound to the entire database that regroups all of the LOPAC molecules. Surprisingly, we found another compound that possesses a very similar structure, differing by a single amino group, but that did not show any inhibitory effect, neither during the original screening nor in the validation assay (Fig 3A and 3B).

### Validation of the Action of S-(4-nitrobenzyl)-6-thioguanosine

**Fig 4:**
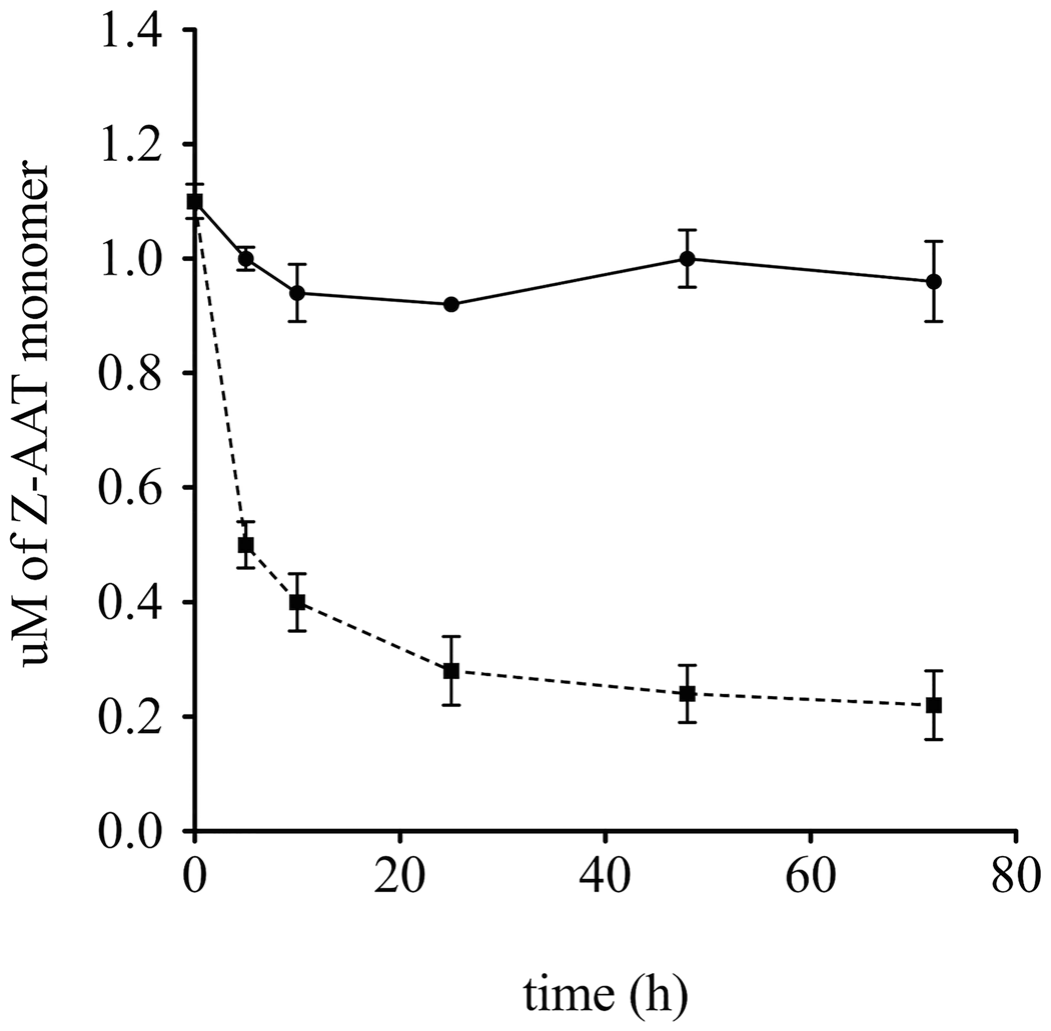
Effect of S-(4-nitrobenzyl)-6-thioguanosine on Z-α1AT polymerization. The protein was incubated with (●) or without (■) 100 μM of S-(4-nitrobenzyl)-6-thioguanosine for various time at 37°C. The error bars are the standard deviation of three separate experiments.

A polymerization reaction was set up in presence or absence of 100 μM of S-(4-nitrobenzyl)-6-thioguanosine and the disappearance of the Z-α1AT monomer monitored by RP-HPLC—a diminution in monomer concentration indicates that the protein has been recruited into polymers. Analysis confirmed that Z-α1AT has its polymerization rate decreased 33 times in presence of the compound and that its effect is long lasting (Fig 4).

**Fig 5:**
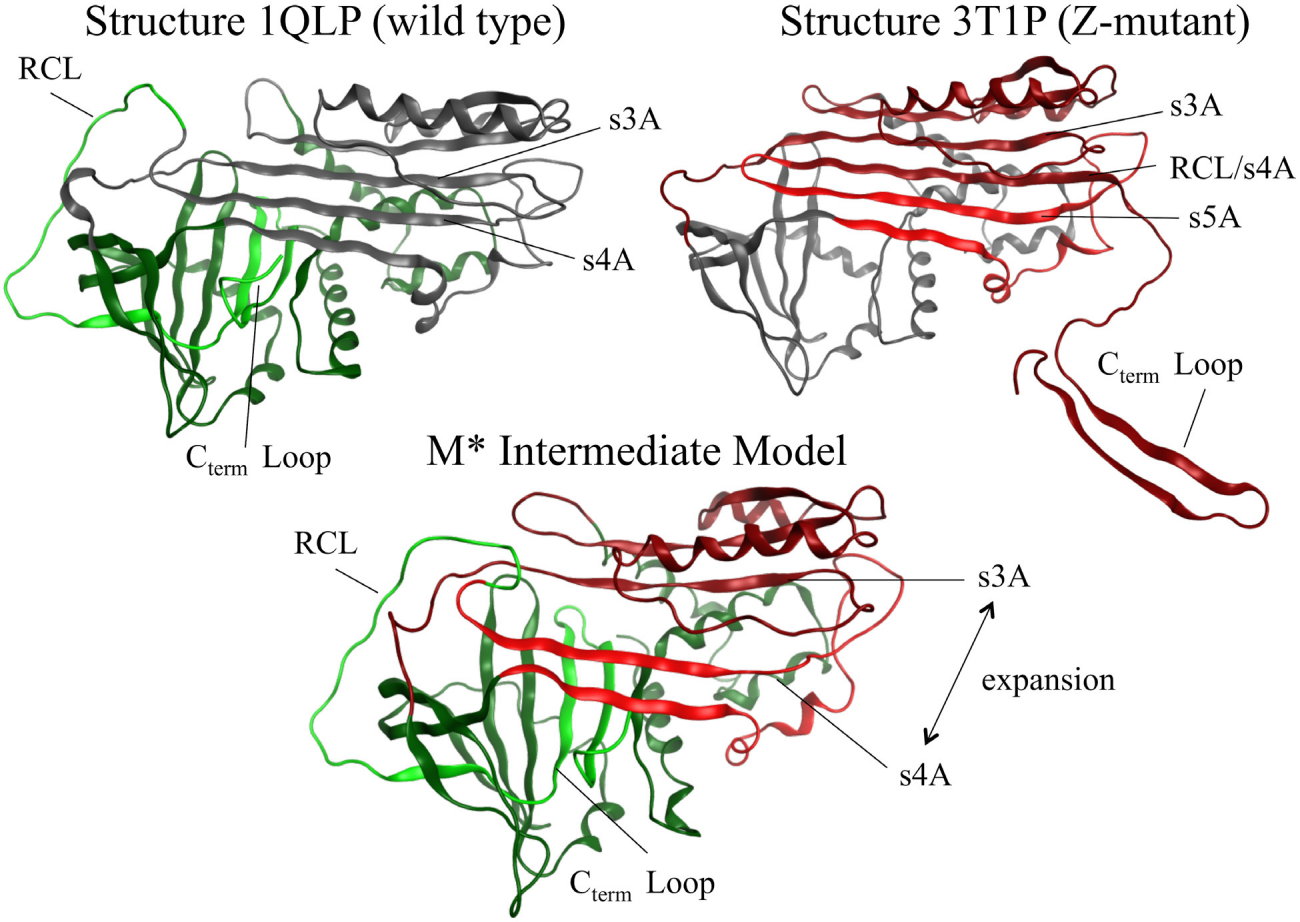
The three models of α1AT protein. (Top left) Structure of wild type (PDB: 1QLP) (Top right) Structure of Z-mutant (PDB: 3T1P) (Bottom middle) Intermediate M* model with an expanded β-sheet A (retained from structure 3T1P), RCL not inserted into the RCL cavity (retained from structure 1QLP), and C_term_ loop inserted into β-sheet B (retained from structure 1QLP). β-sheet A is colored red and β-sheet B is colored green. Shades of green and red distinguish discontinuous fragments from the same initial crystal structure (light/dark green for 1QLP fragments and light/dark red for 3T1P fragments) used to generate the M* model. Grey colored regions represent residues that were not used to generate the M* model.

However, as the s4A cavity does not exist in any crystal structure of α1AT, a theoretical model comparable to M* had to be created. The M* intermediate state is described to have the following three structural features: i) an expanded β-sheet A with a s4A cavity between s3A/s5A, ii) an RCL at the precipice of inserting between s3A/s5A, and iii) the C_term_ loop inserted within β-sheet B and not participating in a domain swap with another protein. These important features of the M* model are represented in the homology model built from the two available crystal structures of α1AT (Fig 5).

**Fig 6:**
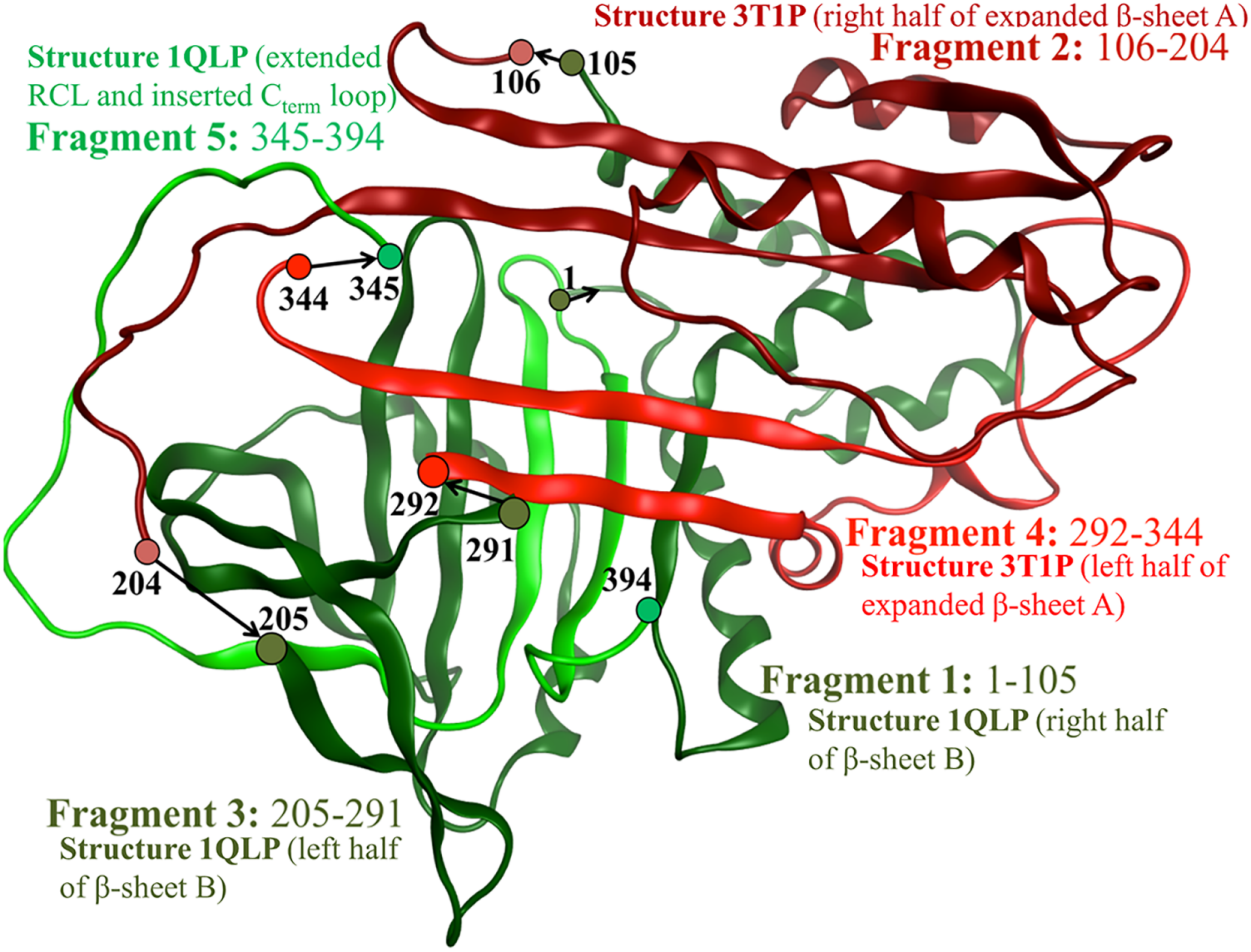
The fragments of structures 1QLP (green) and 3T1P (red) used to homology model the M* intermediate state of α1AT. β-sheet A is the red beta sheet across the top half of the model and β-sheet B is the green beta sheet across the bottom of the model. Residue numbers at the start and end of each fragment transition are labeled with an arrow in the N_term_ to C_term_ direction. Shades of green and red distinguish discontinuous fragments from the same initial crystal structure (light/dark green for 1QLP fragments and light/dark red for 3T1P fragments).

To build the M* state homology model, a total of five protein fragments of the two crystal structures, 1QLP and 3T1P, were merged. Fig 6 shows that fragment 1 consists of residues 1–105 (1QLP) which model the right side of β-sheet B, with respect to beta strands adjacent to the right side of the C_term_ loop. Fragment 3 consists of residues 205–291 which constitute the left side of β-sheet B, with respect to beta strands adjacent to the left side of the C_term_ loop. Together these two fragments model the position of β-sheet B so that the RCL residues from fragment 5 (residues 345–394 from 1QLP) can be placed on the outside of the s4A pocket along with the C_term_ loop buried within β-sheet B. The position of strands s1A, s2A, and s3A in β-sheet A are modeled from fragment 2 (residues 106–204 from 3T1P), and fragment 4 (residues 292–344 from 3T1P) models the position of strands s5A and s6A. Together, the positions of fragments 2, 4 and 5 create the cavity s4A between s3A/s5A, which would otherwise be the site of RCL insertion. This opened conformation of α1AT represents one of the possible structures of the unstable M* intermediate state for which experimental methods such as crystallography cannot reproduce.

### Analysis of α1AT Structures and their Potential Binding Sites

All of the 80 *in vitro* screened compounds, including S-(4-nitrobenzyl)-6-thioguanosine, were docked into every potential binding site to assess if the computational result is comparable to the *in vitro* screening. A binding site able to dock S-(4-nitrobenzyl)-6-thioguanosine with a lower binding energy than the 79 other compounds would be a promising site for further experimental investigations.

**Fig 7:**
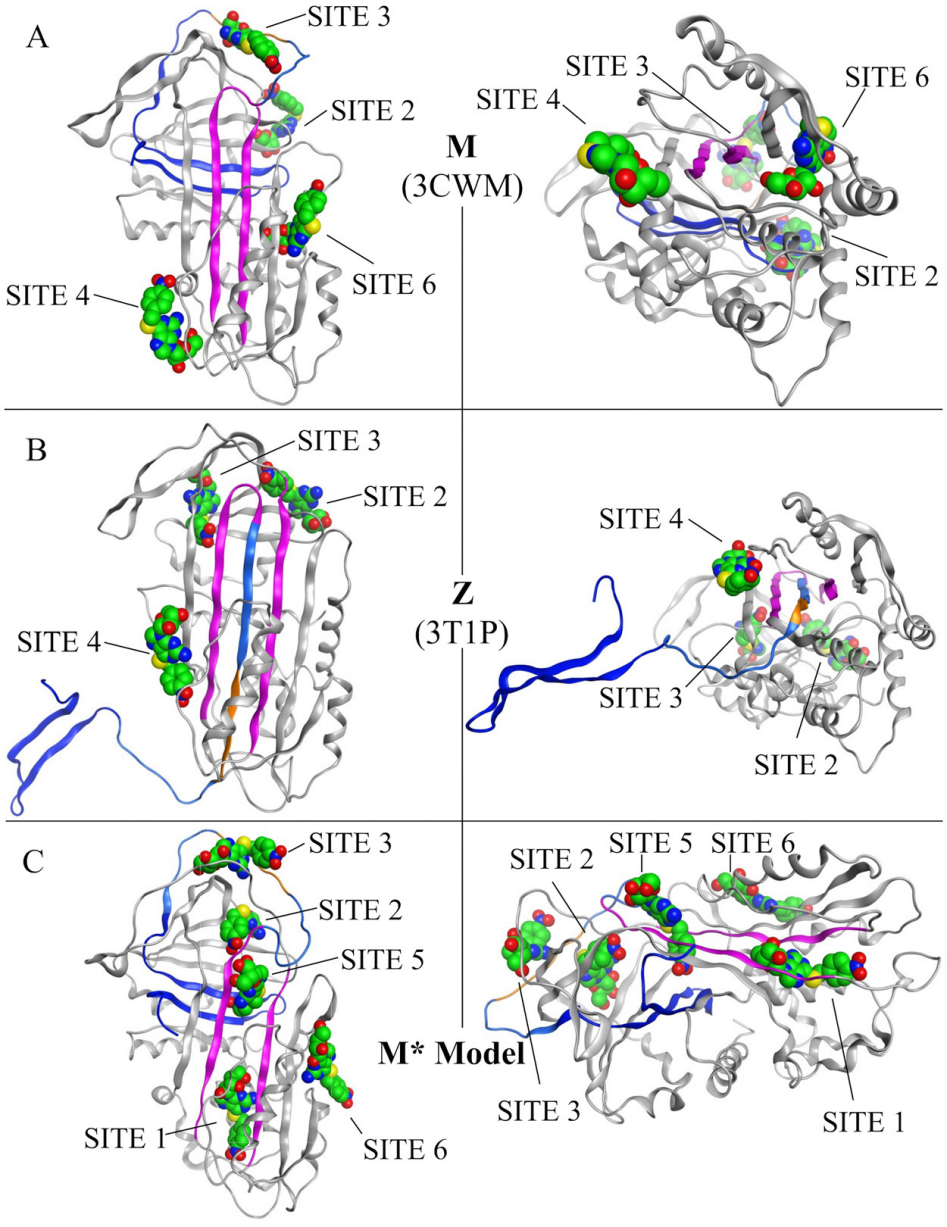
Binding Sites for S-(4-nitrobenzyl)-6-thioguanosine. Two protein ribbon models are shown for each structure: (A) 3CWM, (B) 3T1P and (C) M* Model. The left model and right representations in each panel are rotated 90° with respect to one another. The best binding poses for S-(4-nitrobenzyl)-6-thioguanosine at each available binding site are shown with space filling atoms with the carbon atoms colored green. (Purple) Strands 3 and 5 from β-sheet A. (Dark blue) C_term_ loop within β-sheet B. (Light blue) RCL. (Orange) Residues of the RCL corresponding to the analogous 6-mer peptide.

Six putative binding sites were predicted among the three available protein models: M-α1AT, Z-α1AT and intermediate M* (Fig 7). SITE1 and SITE5 were both exclusively available in the M* model and are located in the RCL insertion site. SITE2 was found in all three models. It is also where the compound citrate, previously reported to lower polymerization rates has been observed to bind in the 3CWM wild type structure [32]. Also found in all three models are: SITE3, a large cavity adjacent to SITE2; SITE4 situated near the C_term_ edge of β-sheet A; and SITE6 located near the N_term_ edge of β-sheet A. A previous work by Gooptu and colleagues have shown that the individual mutations T114F or G117F within SITE6 prevent polymerization without inhibiting protein activity [38]. While SITE6 is present in the three models, it is however partially occluded in our M* model due to the expansion of β-sheet A.

### S-(4-nitrobenzyl)-6-thioguanosine Binds at the RCL Insertion Site or on the Edge of β-sheet A

Docking of all 80 small molecules was performed with each model and at each putative binding site in order to compare how strongly S-(4-nitrobenzyl)-6-thioguanosine binds relative to the 79 other experimentally tested compounds. These results are summarized in Table 1 and present two possible binding sites where S-(4-nitrobenzyl)-6-thioguanosine can favorably bind to block RCL insertion. Results from docking at SITE5, the RCL insertion site, show S-(4-nitrobenzyl)-6-thioguanosine ranking first among the other 79 ligands which may suggest a mechanism where the RCL is directly blocked at the RCL insertion site. Other sites of interest, which rank S-(4-nitrobenzyl)-6-thioguanosine in the top 10% of ligands, are SITE1 and SITE2. SITE1 is also part of the RCL insertion site. SITE2 has been previously reported as the binding site for citrate which can also prevent polymerization and whose mechanism of action has yet to be determined [32]. Interestingly, S-(4-nitrobenzyl)-6-thioguanosine is also found to rank first in the wild type model when docked at SITE6. S1 Fig compares the location of SITE6 in both the M- and Z-α1AT structures which illustrates how binding of S-(4-nitrobenzyl)-6-thioguanosine at SITE6 may prevent the expansion of β-sheet A and possibly prevent RCL insertion.

### Residue Interactions with S-(4-nitrobenzyl)-6-thioguanosine

Nearby residues that interact with S-(4-nitrobenzyl)-6-thioguanosine at the top ranking sites (SITE1, SITE2, SITE5) from the M* intermediate model and the single site (SITE6) from the wild type model are described in Table 2. This information provides the basis for guiding further validations of these binding sites using techniques such as mutagenesis and molecular dynamics. S2–S5 Figs contain additional details about the type of interactions formed between individual atoms of S-(4-nitrobenzyl)-6-thioguanosine and the nearby residues listed in Table 2.

**Table 2:** Residues interacting with S-(4-nitrobenzyl)-6-thioguanosine in top scoring binding sites.

M* Model	SITE1	S34, I35, A37, F38, L41, L149, T157, F159, A160, L161, V162, N163, Y164, L276, F289, L304, K305, L306, K308, A309, V310, H311
	SITE2	W171, E172, R173, P174, F175, R200, M203, F204, N205, L218, M219, K220, Y221, F229, E256, D257, R258, L263, L265, I317, D318, F329, E331
	SITE5	F28, K145, I146, I165, F166, F167, K168, V314, L315, C316, I 317, D318, E319, K320, G321, T322, E323, A324, M351, F361
M-α1AT (3CWM)	SITE6	S56, T59, A60, M63, L100, N104, Q105, L112, T113, T114, G115, N116, G1117, Y138, H139, S140, E141, Y160, G164, N186, Y187, I188

Listed are the interacting residues for binding sites where S-(4-nitrobenzyl)-6-thioguanosine ranks in the top 10% of compounds and less than -7kcal/mol.

## Conclusions

Currently, the only available and effective treatment to correct for the loss of α1AT function in α1ATD associated with liver disease is orthotropic liver transplantation. For lung disease, augmentation therapy is the only specific regiment that is thought to slow down disease progression, although this still requires formal proof through well-controlled clinical trials [39]. As these treatments are expensive, labor intensive and associated with side effects, the need for novel treatments are indeed in high-demand. With Z-α1AT polymerization being responsible for the development of the disease, blocking its aggregation by small molecules [29,32] appears to be a promising strategy to cure Z-α1ATD.

Here we report an integrated *in vitro* and *in silico* approach which allows discovering and characterizing small molecules that disrupt the pathological polymerization of Z-α1AT. The *in vitro* microplate assay, which enables the identification of small molecules able to block the insertion of a modified 6-mer peptide into the s4A cavity, provides quantitative data with reproducibility, sensitivity and rapid throughput. Our results validate the utility of the *in vitro* screening assay and identify S-(4-nitrobenzyl)-6-thioguanosine as inhibitor of Z-α1AT polymerization. With a molecular weight of 434.43 Da, 4 H-bond donors, 11 H-bond acceptors and a low lipophilicity coefficient (XLogP3 = 1.1), this compound presents a drug-like profile according to the Lipinski criteria [40]. From IC_50_ determination and structure-activity relationship studies, we also found one of its structural homologues which differs by a single amino group and does not prevent aggregation. This suggests that an interaction with the amino group may be important to counteract the insertion of the modified 6-mer peptide.

The microplate assay has been designed to identify any inhibitor that can impede the insertion of the RCL into the s4A cavity; compounds may bind at several locations within the s4A cavity or even bind outside of the s4A cavity causing a conformational rearrangement that still precludes RCL insertion. To characterize the binding of S-(4-nitrobenzyl)-6-thioguanosine, molecular docking studies were carried out at several potential binding locations, in and outside the s4A cavity. Previous studies have used molecular docking to investigate the binding of small molecules into experimentally resolved structures at sites other than the s4A cavity [29]. It has been shown [18,41–43] that different lengths of RCL-like peptides, ranging from 4-mer to 13-mer, can be incorporated in place of the RCL to prevent polymerization. The RCL may exhibit different degrees of partial insertion to allow for the remainder of the s4A cavity to be occupied by different lengths of synthetic peptides. The approach used in this paper was to model the fully expanded β-sheet A of Z-α1AT after structure 3T1P which contains a fully inserted RCL and expanded β-sheet A to allow for the use of molecular docking identifying small drug-like molecules that can mimic and compete for the binding state accessed by the RCL across various locations of the s4A cavity. At the same time, sites outside the s4A cavity were investigated using wild type, our M* intermediate model and polymerized mutant structures.

The present development of an atomistic M* model suggests a mechanism through which the newly identified compound S-(4-nitrobenzyl)-6-thioguanosine may inhibit Z-α1AT polymerization by either competing with the RCL at the s4A insertion site (SITE5) or by binding at a nearby location (SITE2) and thus, hindering the s4A cavity.

The part of the cavity that we label as SITE1 may also bind ligands, and may do so with a large stabilizing energy comparable in magnitude to that of SITE5 as indicated in Table 1. The fact that compound B9 is ranked first amongst all the chemicals appears to favor binding in SITE5—assuming that this site can indeed exist, even if only transiently. To definitely discriminate between these binding sites, additional structural studies will need to be carried out beyond the scope of the present work, which will include selected mutagenesis and molecular dynamics (MD). MD simulations, which provide an ensemble of various conformations of M*, will account for the protein flexibility [44,45] and will aid in refining the docking results.

## Supporting Information

S1 Fig Superimposition of structures 1QLP and 3T1P of α1AT to emphasize the disruption of β-sheet A expansion that may occur upon the binding of S-(4-nitrobenzyl)-6-thioguanosine at SITE6. S-(4-nitrobenzyl)-6-thioguanosine is represented with space filling atoms and positioned at SITE6 for the M-α1AT structure (1QLP). (Dark blue) Z-α1AT structure (3T1P) with an expanded β-sheet A. (Dark grey) Wild type structure 3CWM with β-sheet A not expanded into SITE6. (Light blue) expanded β-strand s2A in structure 3T1P, which occupies SITE6. (Light grey) β-strand s2A in structure 1QLP adjacent to SITE6.
(TIFF)

S2 Fig 2-D contour and interaction map generated in MOE for S-(4-Nitrobenzyl)-6-thioguanosine at SITE1 in the M* intermediate state structure. The map shows details about the type of interactions formed between individual atoms of S-(4-nitrobenzyl)-6-thioguanosine and individual atoms of SITE1 of M*.
(TIFF)

S3 Fig 2-D contour and interaction map generated in MOE for S-(4-Nitrobenzyl)-6-thioguanosine at SITE2 in the M* intermediate state structure. The map shows details about the type of interactions formed between individual atoms of S-(4-nitrobenzyl)-6-thioguanosine and individual atoms of SITE2 of M*.
(TIFF)

S4 Fig 2-D contour and interaction map generated in MOE for S-(4-Nitrobenzyl)-6-thioguanosine at SITE5 in the M* intermediate state structure. The map shows details about the type of interactions formed between individual atoms of S-(4-nitrobenzyl)-6-thioguanosine and individual atoms of SITE5 of M*.
(TIFF)

S5 Fig 2-D contour and interaction map generated in MOE for S-(4-Nitrobenzyl)-6-thioguanosine at SITE6 in the 3CWM wild type structure. The map shows details about the type of interactions formed between individual atoms of S-(4-nitrobenzyl)-6-thioguanosine and individual atoms of SITE6 of wild type α1AT.
(TIFF)
