# Supplementary figures and images for: Discovery of an Inhibitor of Z-Alpha1 Antitrypsin Polymerization
Source: PLoS One. 2015 May 11;10(5):e0126256. doi: 10.1371/journal.pone.0126256 (PMC4427445; doi:10.1371/journal.pone.0126256)

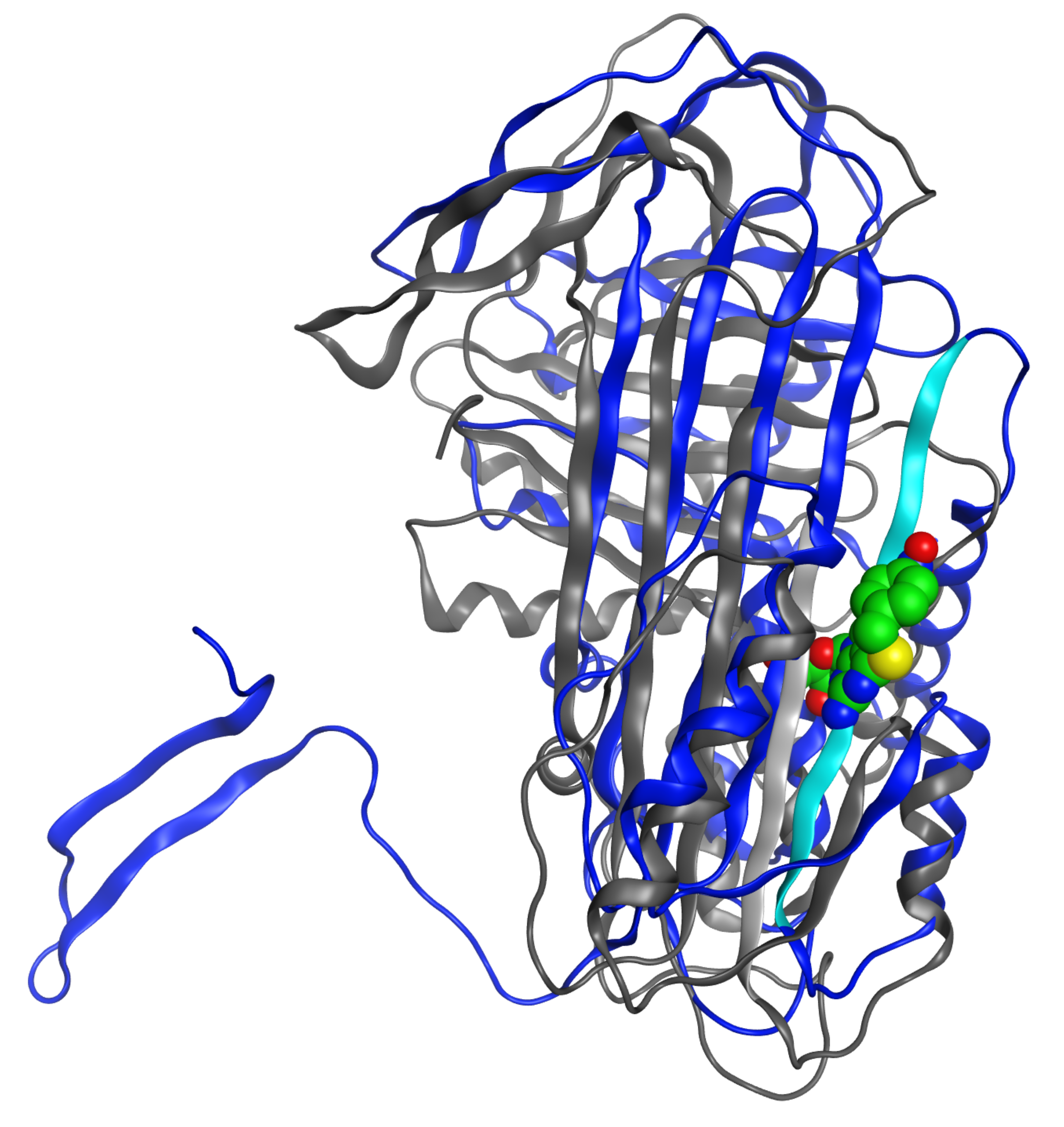

Supplement: S1 Fig — S-(4-nitrobenzyl)-6-thioguanosine is represented with space filling atoms and positioned at SITE6 for the M-α1AT structure (1QLP). (Dark blue) Z-α1AT structure (3T1P) with an expanded β-sheet A. (Dark grey) Wild type structure 3CWM with β-sheet A not expanded into SITE6. (Light blue) expanded β-strand s2A in structure 3T1P, which occupies SITE6. (Light grey) β-strand s2A in structure 1QLP adjacent to SITE6. (TIFF) [file pone.0126256.s001.tiff]

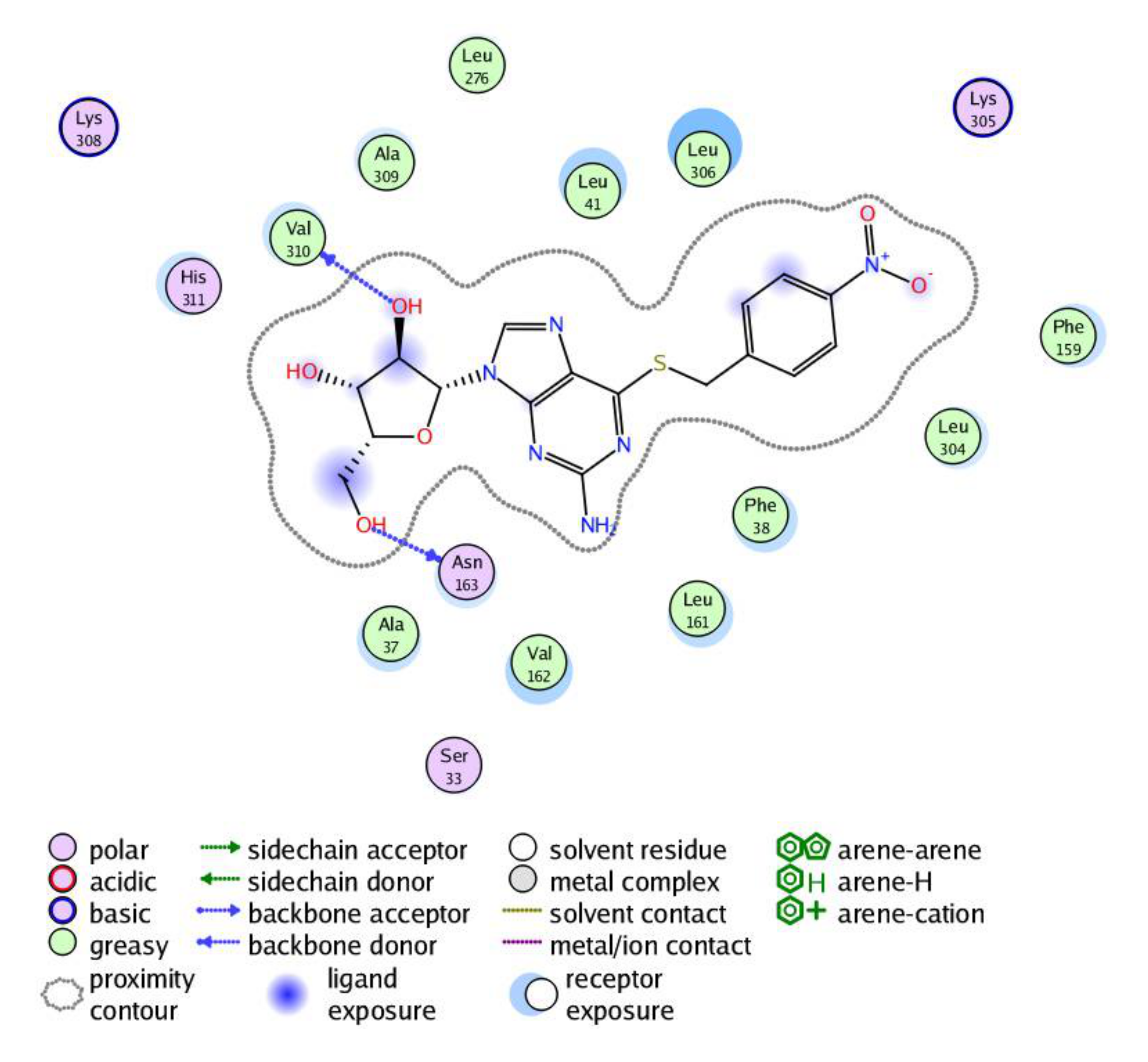

Supplement: S2 Fig — The map shows details about the type of interactions formed between individual atoms of S-(4-nitrobenzyl)-6-thioguanosine and individual atoms of SITE1 of M*. (TIFF) [file pone.0126256.s002.tiff]

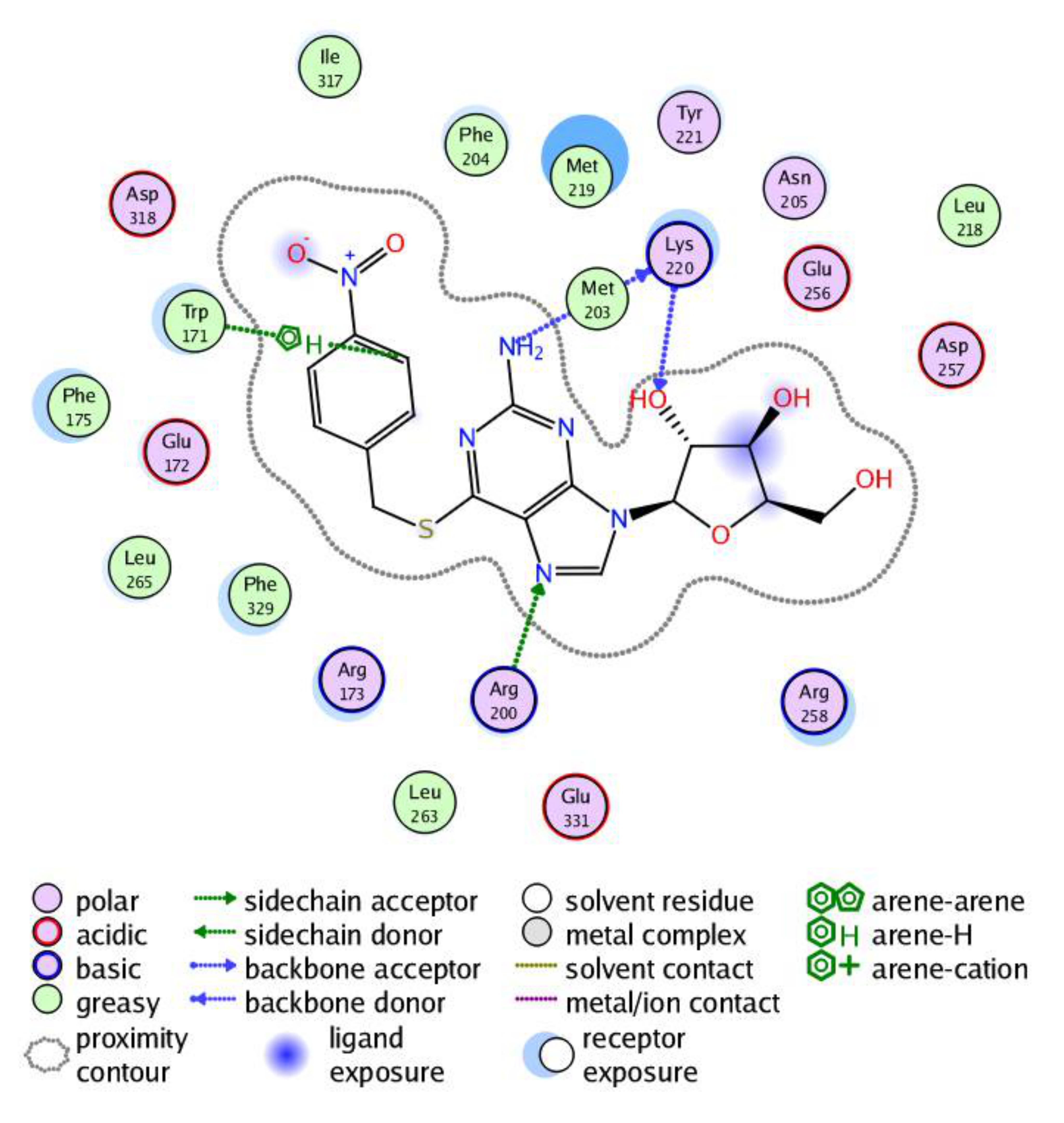

Supplement: S3 Fig — The map shows details about the type of interactions formed between individual atoms of S-(4-nitrobenzyl)-6-thioguanosine and individual atoms of SITE2 of M*. (TIFF) [file pone.0126256.s003.tiff]

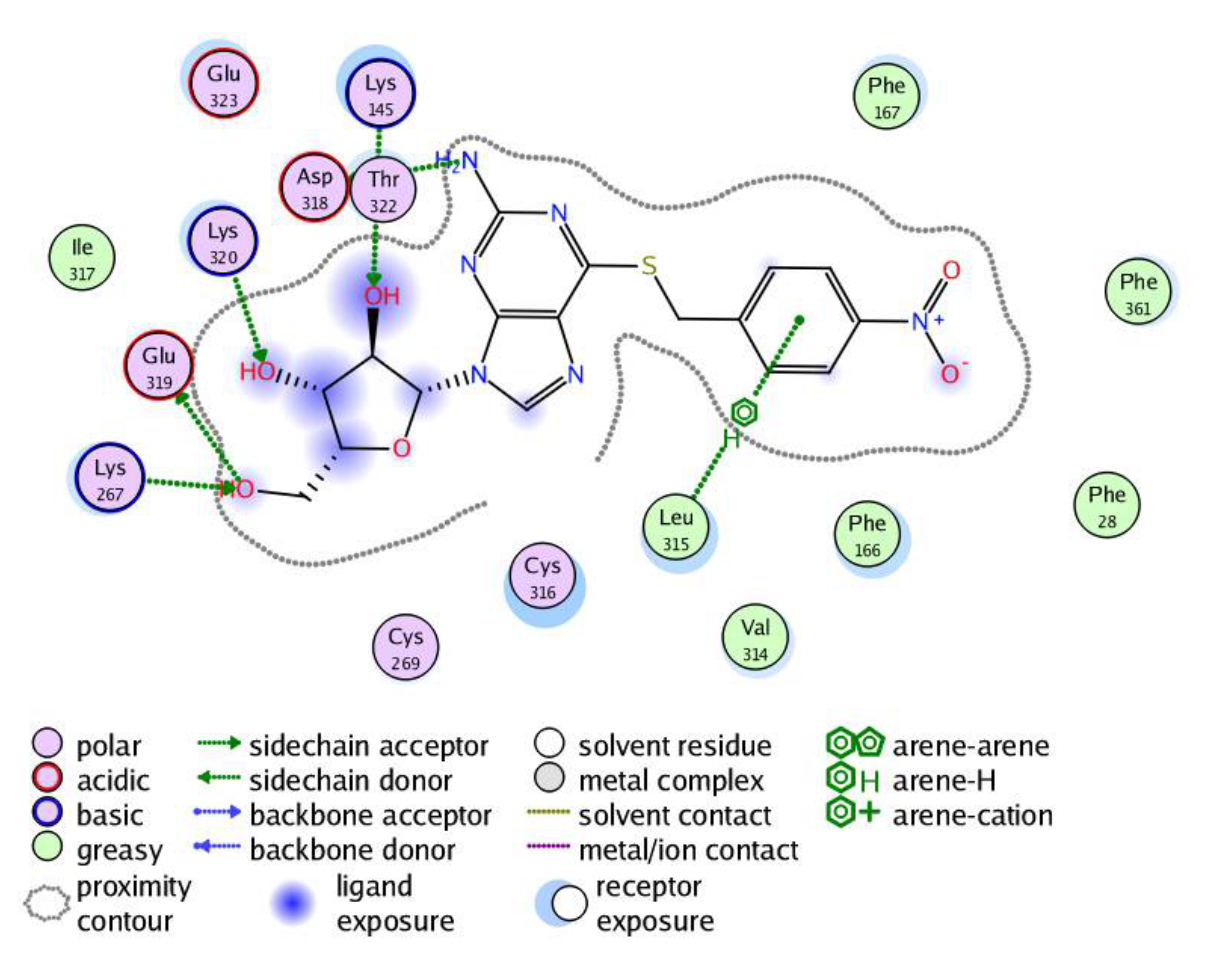

Supplement: S4 Fig — The map shows details about the type of interactions formed between individual atoms of S-(4-nitrobenzyl)-6-thioguanosine and individual atoms of SITE5 of M*. (TIFF) [file pone.0126256.s004.tiff]

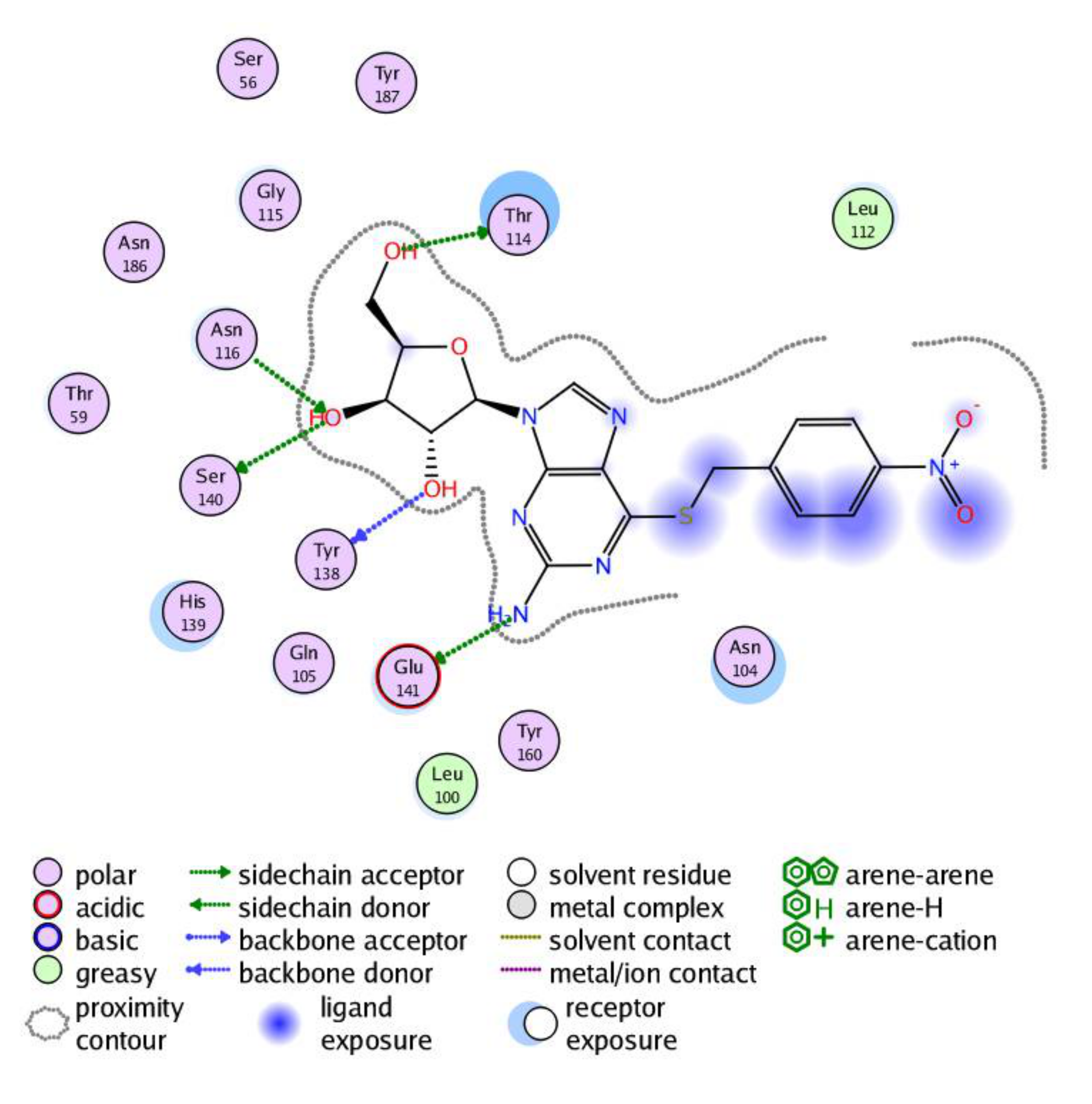

Supplement: S5 Fig — The map shows details about the type of interactions formed between individual atoms of S-(4-nitrobenzyl)-6-thioguanosine and individual atoms of SITE6 of wild type α1AT. (TIFF) [file pone.0126256.s005.tiff]
